# Cardiac Rehabilitation in Heart Failure: Evidence, Coverage, and Implementation

**DOI:** 10.3390/jcm15176576

**Published:** 2026-08-26

**Authors:** Pushan Aggarwal, Aishwarya Yannamani, Jyoti Jain, Ilija Klipa, Andrew Oehler, Hayah Kassis-George

**Affiliations:** 1 Medicine Institute, Allegheny Health Network, Pittsburgh, PA 15212, USA; pushan.aggarwal@ahn.org (P.A.); jyoti.jain@ahn.org (J.J.); 2 Department of Internal Medicine, MedStar Washington Hospital Center, Washington, DC 20010, USA; aishwarya.s.yannamani@medstar.net; 3 Cardiovascular Institute, Allegheny Health Network, Pittsburgh, PA 15212, USA; ilija.klipa@ahn.org (I.K.); andrew.oehler@ahn.org (A.O.)

**Keywords:** cardiac rehabilitation, heart failure, HFrEF, HFpEF, exercise training, insurance coverage, health equity, delivery models, implementation

## Abstract

Cardiac rehabilitation (CR) is a multicomponent secondary prevention intervention that improves functional capacity and quality of life across cardiac diagnoses, yet remains markedly underused in heart failure (HF). Existing reviews treat efficacy, coverage, delivery, and equity separately; this narrative review integrates them to clarify why uptake in HF remains low despite an established evidence base. We searched PubMed/MEDLINE and the Cochrane Library from January 2009 through December 2025, prioritizing clinical practice guidelines, landmark trials, systematic reviews, registry analyses, and U.S. policy documents, with narrative synthesis across efficacy, coverage, delivery, and equity. In heart failure with reduced ejection fraction (HFrEF), exercise-based CR consistently improves peak oxygen consumption by roughly 15 to 17 percent, improves health status, and reduces short-term all-cause hospitalization. In heart failure with preserved ejection fraction (HFpEF), supervised exercise improves functional capacity and quality of life, but a large pragmatic outcome trial and Medicare coverage remain lacking. In appropriately selected, clinically stable patients, hybrid and virtual delivery produce comparable short-term functional and quality-of-life gains, with more limited direct evidence for hard clinical endpoints. Closing the HFpEF coverage gap, automating HFrEF referral, diversifying delivery, and embedding equity across the CR pipeline are actionable priorities for realizing CR’s potential in HF.

## 1. Introduction

Heart failure (HF) affects roughly 6.7 million adults in the United States and is projected to reach more than 8 million by 2030 [1,2]. The condition imposes a substantial burden on patients, families, and health systems through recurrent hospitalizations, progressive functional decline, diminished quality of life, and high mortality. Despite major advances in pharmacological therapy over the past decade, including widespread adoption of sodium–glucose cotransporter-2 (SGLT2) inhibitors, angiotensin receptor–neprilysin inhibitors (ARNIs), and mineralocorticoid receptor antagonists (MRAs), exercise intolerance and symptom burden remain defining features of the disease for most patients [2,3].

Cardiac rehabilitation (CR) is a medically supervised, multidisciplinary secondary prevention program that integrates structured exercise training with risk factor management, education, nutritional counseling, and psychosocial support [4,5]. In the 2022 AHA/ACC/HFSA HF guideline, regular physical activity in patients with HF carries a Class 1 recommendation (Level A) to improve functional status, exercise performance, and quality of life, while referral to a comprehensive cardiac rehabilitation program is designated Class 2a (Level B-R) to improve functional capacity, exercise tolerance, and quality of life [2]. Meta-analytic evidence consistently shows improvements in functional capacity, health-related quality of life, and hospitalization risk in HF patients enrolled in CR [6,7]. The 2024 AHA/AACVPR scientific statement updated the core components of CR to include strength training as a distinct element and introduced program quality as a standalone component, reflecting the recognition that systematic outcomes tracking and quality improvement are integral to program effectiveness rather than optional additions [4].

Despite this evidence base, CR remains one of the most underused guideline-endorsed therapies in cardiovascular medicine. Among Medicare beneficiaries with HF, participation has stayed in the single digits (4 to 8 percent) despite nearly a decade of coverage for heart failure with reduced ejection fraction (HFrEF) [8,9]. Heart failure with preserved ejection fraction (HFpEF), which accounts for about half of all HF cases and is the predominant phenotype among older adults and women, is excluded from Medicare coverage entirely [10,11]. The patients least likely to be referred or to enroll are also the ones with the heaviest disease burden: older adults, women, Black patients, and individuals of lower socioeconomic status [12,13].

The literature addressing CR in HF spans clinical efficacy, coverage policy, delivery model innovation, implementation science, and health equity, but these domains are rarely examined together. Existing systematic reviews and meta-analyses tend to focus narrowly on exercise outcomes or specific populations, while policy and implementation analyses sit in parallel studies. This narrative review attempts to bridge that gap by integrating evidence across all five domains.

Our purpose is to synthesize the current state of evidence on CR in HF across (1) the definition and core components of CR programs, (2) clinical efficacy in HFrEF and HFpEF, (3) insurance coverage and policy, (4) delivery models including telehealth and hybrid approaches, and (5) equity in access and participation. We aim to identify what is well established, what remains uncertain, and where the most pressing evidence and policy gaps lie.

### Literature Search Strategy

We conducted a targeted, non-systematic literature search of PubMed/MEDLINE and the Cochrane Library covering January 2009 through December 2025. The start date was chosen to capture literature from the HF-ACTION trial onward, as HF-ACTION was the primary evidence basis for the 2014 CMS national coverage determination for stable HFrEF and represents a natural inflection point in the field [14]. Searches combined controlled vocabulary (MeSH) and free-text terms across three concept groups joined with the Boolean operator AND: (i) cardiac rehabilitation, exercise-based cardiac rehabilitation, exercise training, exercise therapy, exercise prescription, and physical activity; (ii) heart failure, HF; heart failure with reduced ejection fraction, HFrEF; heart failure with preserved ejection fraction, HFpEF; and heart failure with mildly reduced ejection fraction, HFmrEF; and (iii) selected modifier concepts such as telerehabilitation, home-based, hybrid, coverage, insurance, disparities, and equity. Terms within each group were combined using OR, and results were restricted to English-language human studies. We prioritized clinical practice guidelines, scientific statements, landmark randomized controlled trials, systematic reviews and meta-analyses, individual participant data meta-analyses, large observational cohort and registry-based analyses, and U.S. health policy documents (CMS coverage determinations and federal rulemaking). Reference lists of included articles and pertinent reviews were hand-searched to identify additional sources. Two authors (P.A., A.Y.) independently screened titles and abstracts against relevance criteria. Screening decisions were then compared, and any differences in inclusion were resolved through discussion between the two reviewers. A senior author (H.K.-G.) was designated in advance as adjudicator for any disagreements that could not be resolved by consensus; in practice, all differences were settled at the reviewer level and no items required her adjudication. This review is narrative in scope and does not employ systematic search methodology, formal risk-of-bias assessment, or meta-analytic pooling. Evidence was critically evaluated and synthesized to provide an integrated account of the current landscape and to identify priorities for research and policy.

## 2. What Is Cardiac Rehabilitation?

Cardiac rehabilitation, as defined in the 2024 AHA/AACVPR scientific statement, is a medically supervised, multidisciplinary secondary prevention program that combines structured exercise training, risk factor management, education, and psychosocial support to reduce cardiovascular morbidity and improve functional status and quality of life [4,5]. This definition matters because it makes clear that CR is not simply an exercise program. Multiple controlled trials and meta-analyses confirm that the comprehensive, multicomponent nature of CR (rather than exercise training in isolation) produces the durable improvements in hospitalization risk, functional capacity, and health status observed across cardiac populations [3,6,7].

The 2024 update identifies ten core components: patient assessment, nutritional counseling, weight and body composition management, cardiovascular risk factor management, tobacco cessation, psychosocial management, aerobic exercise training, strength training, physical activity counseling, and program quality [4]. Two aspects of this update deserve emphasis. Strength training was formally recognized as a distinct core component, reflecting substantial evidence that resistance exercise independently combats frailty, preserves functional independence, and improves cardiorespiratory fitness in cardiac populations [4]. Program quality was introduced as a standalone component for the first time, acknowledging that systematic outcomes tracking and quality improvement are essential infrastructure for meaningful gains in CR enrollment, completion, and equity [4,5].

### 2.1. Phases of Cardiac Rehabilitation

CR is organized into three sequential phases, each serving a distinct clinical purpose [3,4].

Phase I (Inpatient) begins during the index hospitalization. The CR team conducts a baseline assessment covering clinical status, functional level, risk factors, cognitive function, psychosocial state, and discharge planning needs [4]. Mobilization is initiated once the patient is clinically stable, which generally requires no new ischemic chest pain in the preceding 8 h, stable or declining cardiac biomarkers, absence of acute decompensated HF signs, and a stable rhythm on telemetry [4]. Exercise at this stage typically consists of supervised walking in short bouts, starting at 3 to 5 min and progressing toward 10 to 15 min as tolerated, with intensity limited to no more than 20 beats per minute above resting heart rate in post-MI patients and 30 beats per minute after cardiac surgery [4]. The primary goals are preventing deconditioning, beginning early patient education, and ensuring a smooth handoff to outpatient CR.

Phase II (Outpatient/Early Recovery) is the structured outpatient program that most clinicians associate with CR. It typically begins within 2 to 12 weeks of discharge and encompasses up to 36 supervised sessions delivered over 36 weeks [4,6]. Each session integrates monitored exercise, individualized exercise progression, risk factor counseling, medication review, and patient education. This phase contains the vast majority of available CR trial evidence, including the foundational HF-ACTION trial [14].

Phase III (Maintenance) follows Phase II and prioritizes long-term exercise adherence and continued risk factor control, generally in less supervised or community-based settings [4]. Long-term adherence remains a consistent challenge. Meta-analytic data confirm that a substantial proportion of HF patients do not sustain structured exercise habits beyond the first year after program completion, with clear implications for the durability of clinical benefit [6,7].

### 2.2. Core Components in Practice

**Exercise prescription.** The 2024 update recommends the FITT-VP framework (frequency, intensity, time, type, volume, progression) for exercise prescription, with iterative adjustment as patients advance through the program [4]. For most cardiac patients in outpatient CR, the standard starting framework involves aerobic exercise three to five days per week at 40 to 80 percent of heart rate reserve or VO2 reserve, for 20 to 60 min per session [4]. When heart rate targets are unreliable, as is often the case in patients with atrial fibrillation or those on high-dose beta-blockade, a rating of perceived exertion (RPE) of 11 to 14 on the Borg 6 to 20 scale serves as an appropriate substitute [4]. Resistance training is typically introduced after several weeks of aerobic training and prescribed at 40 to 60 percent of one-repetition maximum for one to three sets of 8 to 10 exercises on two to three non-consecutive days per week [4].

**Psychosocial assessment and intervention.** Depression, anxiety, social isolation, and low self-efficacy are common in HF and independently associated with poorer CR adherence and worse cardiovascular outcomes [6,7]. Routine screening with validated instruments, combined with targeted counseling, stress management, and mental health referrals when appropriate, is a standard expectation within a well-functioning CR program [4].

**Risk factor modification and education.** CR provides structured, sustained support for smoking cessation, lipid and blood pressure control, glucose management, weight management, and dietary modification [4]. Effective programs pair education with evidence-based behavior change frameworks, including stage-of-change counseling and individualized SMART goal-setting, rather than relying on didactic instruction alone [4].

**Outcomes assessment and quality improvement.** High-functioning programs track clinical, functional, behavioral, and service outcomes and use these data in formal quality improvement (QI) cycles [3,4]. Contemporary meta-analyses confirm that QI interventions, including automatic referral systems, standardized care pathways, and patient navigation, substantially increase CR participation and are now considered part of the core program mandate rather than supplementary activities [7].

## 3. Indications, Eligibility, and Contraindications

### 3.1. Common Indications

CR is a guideline-endorsed intervention across a broad range of cardiovascular conditions, though the class of recommendation varies by indication [2,4]. For patients with HF specifically, the 2022 AHA/ACC/HFSA HF guideline gives regular physical activity a Class 1 recommendation (Level A) and referral to a comprehensive cardiac rehabilitation program a Class 2a recommendation (Level B-R) [2]. Medicare Part B covers CR for patients following myocardial infarction, percutaneous coronary intervention, coronary artery bypass grafting, cardiac valve surgery, heart or heart-lung transplantation, and stable chronic HFrEF [2]. Additional indications supported by guideline evidence but with more variable insurance coverage include peripheral artery disease, adult congenital heart disease, and carefully selected high-risk patients with multiple cardiovascular risk factors who would derive meaningful secondary prevention benefit from medically supervised exercise [3,4].

### 3.2. Heart Failure-Specific Eligibility

For HF patients, the 2022 AHA/ACC/HFSA HF guideline endorses regular physical activity as Class 1 (Level A) and referral to comprehensive cardiac rehabilitation as Class 2a (Level B-R), and it notably does not restrict either recommendation by ejection fraction [2]. In practice, however, Medicare coverage imposes a narrower eligibility standard derived from HF-ACTION and codified in CMS National Coverage Determination 20.31: left ventricular ejection fraction (LVEF) of 35 percent or below, New York Heart Association (NYHA) class II to IV symptoms despite optimized guideline-directed medical therapy (GDMT) for at least six weeks, and clinical stability, defined as no recent (within the preceding six weeks) or planned (within the following six months) major cardiovascular hospitalizations or procedures [14]. HF-ACTION enrolled 2331 medically stable outpatients with systolic HF across 82 centers, and its eligibility criteria became the direct template for the Medicare-covered HFrEF population.

Several additional considerations apply to HF-specific CR readiness. Patients with left ventricular assist devices (LVADs) participate in CR using RPE-based intensity targets of 11 to 13, given the limitations of standard heart rate monitoring in continuous-flow devices [4]. Cardiac resynchronization therapy (CRT) and implantable cardioverter-defibrillator (ICD) recipients require device interrogation and programming review before exercise prescription begins. For ICD patients specifically, exercise intensity must remain at least 10 beats per minute below the device detection threshold [4]. In patients with persistent atrial fibrillation, RPE-based intensity guidance replaces heart rate-based targets throughout the program [2,4].

### 3.3. Contraindications and When to Reconsider

Established contraindications to initiating CR include unstable angina, acute decompensated HF, hemodynamically significant ventricular arrhythmias, high-grade atrioventricular block without pacing, severe symptomatic aortic stenosis or obstructive hypertrophic cardiomyopathy, acute myocarditis or pericarditis, acute pulmonary embolism, uncontrolled hypertension (resting systolic blood pressure above about 180 mmHg or diastolic above 110 mmHg), and severe musculoskeletal conditions that preclude safe exercise [2,4,15]. Most of these are temporary states rather than permanent exclusions. A patient stabilized after acute decompensation who achieves euvolemia on optimized therapy is fundamentally different from one in an ongoing contraindicated state. The 2022 AHA/ACC/HFSA guideline affirms the safety of exercise training across a broad range of clinically stable HF presentations, and reflexively applying contraindication lists as permanent referral barriers contributes to the already low rates of CR utilization in this population [2,15].

## 4. Insurance Coverage and Policy

### 4.1. The Payer Framework Governing CR Access

In the United States, insurance coverage functions as the single most consequential upstream determinant of CR access. Medicare Part B reimburses standard CR programs of up to 36 one-hour sessions, with physician-documented justification allowing extension to 72 sessions, and intensive CR programs of up to 72 sessions over 18 weeks [2]. All programs must be physician-supervised and delivered in a qualifying setting. The 2014 CMS National Coverage Determination that added stable HFrEF to covered CR indications was directly informed by HF-ACTION’s demonstration that supervised exercise training in patients with LVEF of 35 percent or below was safe and produced meaningful improvements in functional capacity and health status [14]. Commercial insurers broadly mirror the Medicare framework, although HFpEF, HFmrEF, stroke, and chemotherapy-related cardiotoxicity are routinely excluded with limited evidence-based justification [2,3].

The 2026 regulatory environment for virtual CR delivery is more nuanced than it initially appears, and conflating the three distinct policies in play creates real practical confusion. The CY 2026 Medicare Physician Fee Schedule Final Rule (CMS-1832-F), finalized on 31 October 2025, permanently authorizes virtual direct supervision of CR, intensive CR, and pulmonary rehabilitation services by physicians and qualified non-physician practitioners via real-time audio-visual communications [16]. That same rule also permanently adds CR, intensive CR, and pulmonary rehabilitation codes to the Medicare telehealth services list, but only for physician office-based programs (POS 11), not for hospital outpatient department (HOPD) settings [16]. Authority for HOPD-based programs to deliver virtual CR to a patient’s home is governed by separate statutory language under Social Security Act §1861(eee)(2)(A)(ii). The Consolidated Appropriations Act of 2026 extended this HOPD home-delivery authority on a temporary basis through 31 December 2027 [17]. The practical implication is that office-based programs have durable virtual delivery authority, while HOPD-based programs (which deliver most CR in the United States) face a statutory cliff at the end of 2027 unless Congress acts. The rule also reaffirmed that non-physician practitioners cannot order CR services, sign treatment plans, or serve as medical directors, maintaining a statutory distinction with practical implications for program staffing and workflow [16].

### 4.2. Structural Consequences of the HFrEF Coverage Rule

The Medicare HFrEF eligibility criteria, although grounded in HF-ACTION’s enrollment framework, create several structural challenges in clinical practice. The requirement for a six-week hospitalization-free window resets entirely with any readmission, creating a cycle in which the highest-risk HFrEF patients (also the most likely to decompensate) are repeatedly pushed out of eligibility [8,9]. Unlike post-MI or post-CABG referrals, which can be embedded in discharge order sets as single-criterion triggers, HFrEF CR referrals require manual verification of LVEF, NYHA class, medication optimization status, and the temporal eligibility window. This added documentation layer suppresses referral rates in high-volume or resource-limited settings [9,18].

Real-world data confirm the consequences. Registry-based analyses show that fewer than one in three eligible HFrEF patients discharged from hospital receive a CR referral, and among Medicare beneficiaries, CR participation rates have remained in the 4 to 8 percent range despite nearly a decade of coverage [8,9]. Patients who are not referred are disproportionately older, female, Black, and more medically complex, a pattern that reflects structural inequities embedded in the current referral infrastructure rather than clinical unsuitability for CR [12].

### 4.3. HFpEF: The Most Consequential Coverage Gap

HFpEF accounts for approximately half of all HF cases and is the predominant phenotype among older adults and women, yet it remains excluded from Medicare CR coverage as of early 2026 [10,11]. This exclusion has measurable downstream effects. Programs frequently decline to enroll HFpEF patients even when referred, anticipating reimbursement denial, and clinicians in turn withhold referrals in anticipation of coverage rejection. The result is a self-reinforcing cycle of non-access [8,9]. The available randomized trial evidence and systematic reviews now consistently show that exercise training improves peak VO2 and health-related quality of life in HFpEF, and the 2023 AHA/ACC scientific statement on supervised exercise in HFpEF explicitly endorsed its clinical benefit [19]. The AHA has formally called for CMS coverage expansion to include stable HFpEF, and coordinated advocacy and research efforts are underway to build the pragmatic evidence base needed to support a formal coverage determination [5,19].

### 4.4. Practical Implications for Clinicians

For the practicing clinician, the coverage environment demands active navigation. HFrEF CR referral documentation must confirm LVEF of 35 percent or below, NYHA class II to IV symptoms, at least six weeks on optimized GDMT, and clinical stability, defined as no major cardiovascular hospitalization or procedure in the preceding six weeks and none planned within the following six months [2,14]. Incomplete documentation is a preventable and common source of denied authorizations. For post-MI, post-CABG, and post-PCI patients, EHR-embedded discharge order sets automate referral and have been shown to substantially increase referral rates when combined with patient navigation [8,15]. For HFpEF patients, clinicians should consider appealing coverage denials with documentation of functional impairment and citation of the AHA/ACC 2023 scientific statement [19]. Programs should also actively track the evolving policy landscape around HFpEF coverage expansion, as any such expansion would require meaningful adjustments in program capacity, staffing, and delivery model planning [4,5].

## 5. Outcomes and Metrics for Assessing CR Efficacy

### 5.1. Functional Capacity: CPET and the Six-Minute Walk Test

Cardiopulmonary exercise testing (CPET) with peak VO2 measurement remains the gold standard for quantifying functional capacity in HF and carries both prognostic and interventional significance [3,14]. In HFrEF, the mechanisms of exercise intolerance are multifactorial and include impaired peak cardiac output, attenuated peripheral vasodilation, skeletal muscle hypoperfusion, and reduced mitochondrial oxidative capacity, which together reduce exercise tolerance to roughly 40 percent of age- and sex-matched controls [10,14]. The ventilatory efficiency slope (VE/VCO2) classifies HF patients as high risk when it exceeds 34 to 36 and provides independent prognostic information beyond peak VO2 alone [3]. A key sub-analysis of HF-ACTION showed that each 6 percent relative improvement in peak VO2 with exercise training was independently associated with a 5 percent reduction in all-cause mortality or hospitalization and a 7 percent reduction in all-cause mortality [14].

Meta-analyses consistently show that CR produces a 15 to 17 percent improvement in peak VO2 in HFrEF populations [6,20]. An individual participant data meta-analysis confirmed clinically important benefits across patient subgroups, including older adults and those with more advanced HF symptoms [21]. A 2025 systematic review and meta-analysis of telehealth-based CR in HF found that remote delivery also produced significant improvements in six-minute walk distance (standardized mean difference [SMD] 0.35; 95% CI 0.15 to 0.55; *p* < 0.001) relative to usual care [22]. The six-minute walk test (6MWT) is the most practical functional assessment tool for routine CR use, correlating well with peak VO2 and demonstrating excellent test–retest reliability [20,23]. The Short Physical Performance Battery (SPPB) and 30-s sit-to-stand test complement aerobic capacity measures by capturing balance, gait speed, and lower-extremity strength, domains of functional limitation that peak VO2 does not address [4].

### 5.2. Patient-Reported Outcomes and Quality of Life

Health-related quality of life is a primary patient-centered outcome in CR for HF, where symptom burden and functional limitation often have the largest day-to-day impact on patients’ lives. The Kansas City Cardiomyopathy Questionnaire (KCCQ) is the most validated HF-specific patient-reported outcome instrument and is increasingly specified as a co-primary endpoint in HF trials and CR program evaluations [3,24]. In HF-ACTION, exercise training produced significant and clinically meaningful improvements across all KCCQ domains within three months, with benefits sustained throughout follow-up; the number needed to treat for a clinically meaningful KCCQ improvement was 4 at three months [24]. A systematic review of KCCQ use in CR settings (9 studies, 3095 patients) confirmed that moderate-to-high-intensity exercise training consistently improved KCCQ scores, with the largest effects observed in high-intensity interval training (HIIT) protocols [3,24].

### 5.3. Hospitalization and Utilization Outcomes

The 2024 Cochrane systematic review and meta-analysis of exercise-based CR in HF, encompassing 60 trials and 8728 participants, found that CR significantly reduced all-cause hospitalizations in the short term (risk ratio [RR] 0.69; 95% CI 0.56 to 0.86; number needed to treat [NNT] 13) relative to usual care [7]. A large retrospective cohort study found 26 percent lower odds of hospitalization at two years among CR participants compared with non-participants, with an all-cause mortality benefit that strengthened with longer follow-up [25]. These findings reinforce the point that the benefit of CR is proportional to what is actually delivered and received, not simply to enrollment [6,7].

### 5.4. Program-Level Metrics

The referral rate approximates 25 percent in HF populations against a recognized target of 70 percent [8,9]. Enrollment lags behind referral due to failures in the post-discharge interval [9,18]. Adherence and completion rates in HF populations are lower than in other cardiac diagnoses, with a meaningful proportion of enrolled patients discontinuing before completing the full prescribed course [7]. Programs certified through AACVPR report outcomes to a national registry that enables risk-adjusted benchmarking and supports quality improvement cycles targeting these persistent gaps [4].

## 6. Evidence Summary in HFrEF

The evidence base for CR in HFrEF is substantial and shows consistent improvements in functional capacity and health-related quality of life, with more variable effects on hard clinical endpoints [7].

The landmark HF-ACTION trial enrolled 2331 patients with systolic HF (LVEF 35 percent or below, NYHA class II to IV) and randomized them to supervised exercise training plus usual care versus usual care alone [14]. The trial did not meet its primary endpoint on intention-to-treat analysis, but after prespecified adjustment for prognostic baseline variables, it showed an 11 percent reduction in all-cause mortality or hospitalization (hazard ratio 0.89; *p* = 0.03) and a 15 percent reduction in cardiovascular mortality or HF hospitalization (hazard ratio 0.85; *p* = 0.03) [14]. These adjusted results were consistent with the broader meta-analytic literature and have been interpreted as reflecting the challenges of maintaining exercise adherence in a real-world trial context, rather than an absence of biological effect.

Meta-analyses consistently report that exercise-based CR improves peak VO2 by approximately 15 to 17 percent and enhances six-minute walk distance, exercise duration, and workload [6]. The 2019 individual participant meta-analysis confirmed clinically important benefits in exercise capacity across patient subgroups [21]. A subsequent systematic review found significant improvements in both six-minute walk distance (SMD 0.30) and oxygen consumption (SMD 0.23) with comprehensive CR [20]. The 2024 Cochrane review reported that CR improved health-related quality of life measured by the Minnesota Living with Heart Failure Questionnaire (MLHFQ) by a mean of −7.39 points (95% CI −10.30 to −4.47), exceeding the 5-point threshold for clinical importance (lower MLHFQ scores indicate better quality of life) [7]. Most meta-analyses show no significant short-term mortality benefit (RR 0.93; 95% CI 0.71 to 1.21), although more recent analyses suggest that mortality benefits may emerge with longer follow-up [6,7].

CR reduces all-cause hospitalizations in the short term (RR 0.69; 95% CI 0.56 to 0.86), and a retrospective cohort study found 26 percent lower odds of hospitalization at two years among CR participants [7,25]. In a cohort of 772 HF patients completing a 3-month program, completion was independently associated with 34 to 44 percent relative risk reductions in mortality and HF hospitalization compared with non-completers [26]. A 2024 network meta-analysis found that combined aerobic and resistance training was the most effective modality for improving quality of life, exercise capacity, and reducing rehospitalizations in chronic HF [27]. CR also supports pharmacological adherence; a multicenter German HFrEF cohort showed systematic optimization and high ongoing use of guideline-directed drugs, including SGLT2 inhibitors and sacubitril/valsartan, maintained over six months [28].

Despite this evidence and Medicare coverage, real-world participation remains strikingly low, with only 4 to 8 percent of eligible patients attending at least one session [8].

## 7. HFrEF Under-Referral and Low Participation

The most useful way to frame CR underutilization is as a pipeline problem in which eligible patients drop out at each sequential stage: eligibility identification, referral placement, enrollment, adherence, and completion. Registry and claims-based studies show that fewer than one in three eligible HFrEF patients discharged from hospital receive a CR referral, and among Medicare beneficiaries, participation rates have remained in the single digits despite coverage being available since 2014 [8,9]. Women, older adults, Black patients, and those with greater comorbidity burden are underrepresented at every stage, even after accounting for clinical complexity [12].

The pipeline breaks for identifiable, addressable reasons. Confirming LVEF of 35 percent or below, NYHA class II to IV, adequate time on GDMT, and the six-week hospitalization-free window requires manual chart review that is difficult to automate in most electronic health record (EHR) systems [9,18]. Among patients who are referred, many never attend a first session. The post-discharge interval is a particularly vulnerable period where lack of follow-up contact, insurance confusion, and logistical barriers compound to prevent enrollment [8,15]. Transportation challenges, distance to CR facilities, work and caregiving obligations, copayment burden, depression, and limited understanding of what CR actually involves are all documented barriers that disproportionately affect the populations already least likely to be referred [12,13,18].

### 7.1. Strategies That Improve the Pipeline

**Automatic referral.** EHR-embedded discharge order sets that trigger a CR referral without requiring a separate physician action have been shown to dramatically increase referral rates across cardiac populations [8,15]. For HFrEF, semi-automated clinical decision support tools that flag eligible patients and prompt the ordering provider have shown meaningful referral rate improvements in early implementation studies [9].

**Patient navigation.** A dedicated CR navigator embedded in the inpatient HF service, responsible for identifying eligible patients, explaining CR at the bedside, arranging the first appointment before discharge, and troubleshooting barriers post-discharge, addresses multiple pipeline failures at once [3,15]. Combining automatic referral with navigation achieves higher enrollment rates than either strategy alone [15].

**Early scheduling.** Scheduling the first outpatient CR session before hospital discharge, rather than leaving coordination to the patient afterward, substantially reduces attrition in the post-discharge interval [8,9].

**Hybrid and remote delivery.** Removing the requirement for in-person attendance eliminates the most common structural barriers to enrollment and completion. Telehealth-based CR in HF has demonstrated adherence rates of 70 to 92 percent and functional improvements comparable to center-based CR across 14 randomized trials [22].

**Performance feedback.** Providing referring teams with regular, benchmarked data on their patients’ CR referral, enrollment, and completion rates reinforces accountability and has been incorporated into high-performing quality improvement programs [4].

### 7.2. HFmrEF: A Coverage and Evidence Blind Spot

Heart failure with mildly reduced ejection fraction (HFmrEF; LVEF 41 to 49 percent) sits in a category deliberately created by the 2021 universal HF definition and formally endorsed in the 2022 AHA/ACC/HFSA HF guideline, which recommends that HFmrEF patients be treated similarly to those with HFrEF with GDMT [2,29]. For exercise-based rehabilitation, the guideline treatment framework carries directly across the reduced and mildly reduced categories, and pooled analyses of exercise training in HFrEF have generally included patients with LVEF in the low forties without evidence of a threshold effect [6,21]. Trial-level data specifically restricted to HFmrEF cohorts are limited, but small prospective studies and secondary analyses of contemporary HF exercise trials suggest that improvements in peak VO2, six-minute walk distance, and KCCQ scores in HFmrEF are comparable to those observed in HFrEF, supporting the biological plausibility of a shared training effect [3,6,21]. Despite this, CMS coverage under NCD 20.31 requires an LVEF of 35 percent or below, leaving HFmrEF patients formally outside the covered stable HFrEF population and, in most commercial plans, without a discrete covered indication [2,14]. Program leaders and clinicians should be aware that HFmrEF is likely to be included in any future CMS coverage reconsideration in HF, and pragmatic trials designed to inform an HFpEF coverage determination should where possible enroll HFmrEF patients as a co-primary or prespecified subgroup population, allowing simultaneous evidence generation across the LVEF spectrum currently outside coverage.

## 8. Evidence Summary in HFpEF

Exercise-based CR in HFpEF produces consistent short-term benefits in functional capacity and quality of life, but heterogeneity in study populations and intervention design, combined with limited durability data, makes a single uniform protocol approach inadequate for this population [30].

Structured exercise in HFpEF (across aerobic, resistance, inspiratory muscle training, and supervised CR formats) leads to significant gains in peak VO2 and six-minute walk distance [30]. Fukuta and colleagues, in a meta-analysis of five randomized exercise trials totaling 245 patients, found that exercise training improved peak VO2 by 2.3 mL/min/kg, six-minute walk distance by 30 m, and MLHFQ scores by 9 points compared with usual care [30]. Quality of life improves meaningfully across disease-specific and generic instruments, including the MLHFQ, KCCQ, SF-36, and EQ-5D [31,32].

Mapping training modalities to the dominant physiological limitations in HFpEF clarifies why a single protocol is unlikely to serve all patients equally. Exercise intolerance in HFpEF arises predominantly from peripheral rather than central limitations: impaired peripheral oxygen extraction and skeletal muscle oxidative capacity, blunted cardiac output augmentation on effort (frequently reflecting chronotropic incompetence), and exaggerated exercise-induced elevations in left ventricular filling pressure with associated pulmonary vascular reserve limitation [33,34]. Aerobic training targets peripheral oxygen extraction, mitochondrial oxidative capacity, and endothelial function, and it is the modality most consistently associated with gains in peak VO2 across HFpEF trials [30,33]. Resistance training targets skeletal muscle mass, strength, and functional performance, and it is particularly important in the frail and sarcopenic phenotype, where it complements aerobic gains and improves ability to perform activities of daily living [33,35]. Inspiratory muscle training addresses respiratory muscle weakness that contributes to dyspnea on effort and can improve functional capacity in patients with reduced inspiratory strength [33]. High-intensity interval training may produce larger peak VO2 gains than moderate continuous training in appropriately selected, clinically stable patients, but tolerability is more limited in older and frailer HFpEF phenotypes, and OptimEx-Clin showed that these gains did not persist once supervised training ended [36]. In a phenotype-informed framework, the metabolic/obese phenotype benefits most from combined aerobic and resistance training paired with weight-loss strategies; the frail elderly phenotype benefits from lower-intensity, multidomain rehabilitation emphasizing strength, balance, and gait, as in REHAB-HF; the atrial fibrillation-predominant phenotype requires rate and rhythm optimization with RPE-guided intensity; and the pulmonary hypertension-predominant phenotype requires careful screening and, in most cases, initiation in a center-based setting with individualized progression [33,34,37]. On a mechanistic level, exercise training in HFpEF enhances skeletal muscle function and improves inflammatory profiles, with some studies showing improvements in diastolic indices such as E/e′, although structural cardiac reverse remodeling remains inconsistent across studies [33]. Long-term effects on mortality and HF hospitalization remain unproven, making large outcome-driven trials an important research priority [33].

### HFpEF Phenotypes and the Limits of a Single-Program Approach

Multiple lines of evidence underscore that HFpEF is a multisystem and phenotypically diverse syndrome, and that a one-size-fits-all rehabilitation program is unlikely to serve the full spectrum of patients equally [38,39]. Systematic reviews identify several recurring phenotypes: metabolic or obese, atrial fibrillation-dominant, frail elderly, cardiorenal–metabolic, pulmonary hypertension-predominant, and younger patients with low comorbidity burden [38].

The most prevalent HFpEF phenotype is metabolic, with over 80 percent of patients having overweight or obesity, metabolic derangements, and impaired skeletal muscle function [19]. Combined exercise and diet-induced weight loss shows particular promise in this group; modest weight loss of approximately 6.6 percent through caloric restriction improved peak VO2 by 1.3 mL/kg/min in a key trial [11]. Frailty-dominant HFpEF is characterized by older patients with sarcopenia and physical function impairment across multiple domains. These patients benefit more from multidomain interventions that include balance training, gait training, and comprehensive geriatric assessment than from aerobic training alone, as the REHAB-HF trial demonstrated [37]. The atrial fibrillation-associated phenotype requires careful rate and rhythm optimization, RPE-guided exercise intensity, and an emphasis on lower-intensity endurance combined with balance and resistance components to address frailty and fall risk [33,39].

Approximately one-third of HFpEF patients are non-responders to standard exercise programs, and the specific cardiac versus peripheral adaptations vary by disease stage and dominant physiological limitation [34]. Precision approaches that use phenotype-guided program design represent the next frontier for optimizing exercise-based therapy in this complex and growing population [38,39,40].

## 9. The HFpEF Evidence-to-Coverage Gap

### 9.1. Why Coverage Has Lagged

The absence of Medicare coverage for HFpEF reflects three converging factors. HFpEF spans multiple pathophysiologic phenotypes, each with different exercise physiology, comorbidity profiles, and training responses, making it difficult to generate the uniform efficacy signal that coverage determinations typically require [38,39,40]. There is also no trial analogous to HF-ACTION that provides multicenter scale, comprehensive safety documentation, and a measurable effect on a composite of mortality and hospitalization in a HFpEF population [14]. The available HFpEF trials are smaller, more heterogeneous in design, and predominantly report functional endpoints rather than hard clinical outcomes [30,31,32]. Finally, inconsistent reporting of intervention dose across HFpEF exercise trials has made it difficult for payers to determine whether a given trial’s intervention resembles the program they would be asked to cover [19,33].

### 9.2. What Coverage-Grade HFpEF Evidence Should Look Like

Future HFpEF trials designed to inform coverage policy must describe their intervention in terms that map directly to a standard CR program: 36 supervised sessions over up to 36 weeks, delivered in an approved setting under physician oversight, incorporating all AHA/AACVPR core components, with transparent reporting of session frequency, duration, exercise intensity, modalities used, and actual adherence [4,14]. The endpoint framework should pair the KCCQ as the primary patient-reported outcome (minimum clinically important difference of 5 points on the Clinical Summary Score) with functional endpoints, including peak VO2 where feasible, and pragmatic alternatives such as the 6MWT, SPPB, and 30-s sit-to-stand test [4,24]. HF-related and all-cause hospitalization should be pre-specified secondary outcomes with at least 12 months of follow-up, and durability assessments at 6, 12, and 24 months are needed given the OptimEx-Clin finding that functional gains did not persist after supervised training ended [36]. Enrolling patients from community hospitals, safety-net health systems, and rural settings, and incorporating hybrid and telehealth delivery alongside center-based programs, will strengthen generalizability and policy relevance [22,33]. The FUNNEL+ study protocol, which prospectively examines a comprehensive CR program in elderly HFpEF patients using imaging and biomechanical biomarkers, illustrates the kind of design needed to move the field forward [41]. Pre-specified subgroup analyses by HFpEF phenotype, sex, and race or ethnicity are essential for interpreting treatment response heterogeneity [38,39].

### 9.3. Coverage and Equity Must Be Addressed Together

Any coverage expansion for HFpEF that does not simultaneously address access barriers risks extending coverage in name while failing to reach the patients who need it most. HFpEF disproportionately affects older women, Black patients, and individuals with obesity, diabetes, and hypertension, the same populations that already experience the largest CR access disparities [12,13,42]. Copayment mitigation, transportation access, and culturally appropriate program delivery are prerequisites for equity, not peripheral considerations [13].

## 10. CR Delivery Models

### 10.1. Defining the Delivery Spectrum

Traditional CR is conducted in a supervised, center-based setting where patients attend scheduled sessions at a hospital outpatient facility or approved physician office, with direct monitoring, telemetry as needed, and an in-person multidisciplinary team [4,7]. This model remains the reference standard, particularly for higher-risk HF patients where direct monitoring and rapid clinical response capability matter most. A hybrid model blends early in-person sessions with subsequent remote or home-based exercise [6,22]. Virtual CR uses synchronous video-based supervised exercise, while fully remote or home-based programs rely on asynchronous monitoring, digital coaching, and structured self-management with periodic clinician contact [22,43]. The distinguishing features and supervision intensity of these delivery models are summarized in Table 1.

### 10.2. Evidence Across Delivery Models

For high-risk HF patients with complex arrhythmia histories, recent ICD therapy, LVAD dependence, or severe functional limitation, the safety margin of center-based CR is clinically meaningful and difficult to replicate remotely [3,4]. However, a 2025 systematic review and meta-analysis of randomized trials of telehealth CR in HF found that remote delivery significantly improved six-minute walk distance (SMD 0.35; 95% CI 0.15 to 0.55) and health-related quality of life relative to usual care, with adherence rates of 70 to 92 percent and consistently high patient satisfaction [22]. A broader 2026 narrative review of cardiac telerehabilitation and digital health frontiers likewise concluded that cardiac telerehabilitation is non-inferior to center-based programs for exercise capacity and quality of life in appropriately selected patients, most robustly in stable, non-frail, digitally literate individuals without unstable arrhythmias, while cautioning that evidence for hard clinical endpoints, higher-risk populations, and long-term durability remains limited [43]. A network meta-analysis across delivery modes found that combined aerobic and resistance training, regardless of delivery setting, produced the most consistent benefits on quality of life and exercise capacity in chronic HF [27]. Delivery model selection should therefore be guided by patient risk profile, access barriers, and logistical feasibility rather than a blanket assumption that center-based programs are superior for all stable, lower-risk patients [7,22,43]. Coverage architectures for virtual delivery also differ across health systems; in single-payer and mixed-financing systems in Europe, Australia, and parts of Asia, cardiac telerehabilitation is increasingly incorporated into national quality frameworks (including as a European Association of Preventive Cardiology accreditation indicator), whereas the U.S. reimbursement pathway remains bifurcated between office-based and hospital outpatient department settings and dependent on periodic legislative extensions [16,17,43].

### 10.3. Monitoring, Safety, and Escalation

For HF patients in telehealth- or home-based CR, key safety elements include a structured pre-session symptom screen, remote vital sign monitoring with data transmitted to the clinical team, defined escalation thresholds (such as weight gain of more than 2 to 3 pounds in 24 to 48 h or resting oxygen saturation below 90 percent), and a written emergency action plan reviewed with patients and caregivers before enrollment [2,4]. ICD patients in remote programs require intensity prescriptions calibrated to remain at least 10 beats per minute below the device detection threshold, with any device activation triggering immediate suspension of exercise and clinical contact [4]. Higher-risk patients with complex arrhythmias, recent ICD therapy, LVAD dependence, or advanced pulmonary hypertension are best served by center-based programs, at least initially [2,3].

### 10.4. Implementation Considerations

Staff must build competency in digital health platforms, remote monitoring interpretation, and telehealth communication on top of core CR clinical expertise [4]. EHR workflows should capture remote session data (exercise logs, vital signs, symptom reports, and patient-reported outcome survey results) in a structured format integrated with the patient’s primary record and AACVPR registry reporting requirements [4]. Standardized pre-enrollment assessments of home internet access, device availability, and digital confidence, paired with provision of equipment or connectivity support where needed, are practical steps toward ensuring telehealth CR reaches the patients who stand to benefit most from its flexibility [12,22].

## 11. Equity: Women, Racial Disparities, and Social Determinants of Health

Disparities in CR participation appear across the entire care continuum. Only about 25 percent of HF patients with reduced EF are referred, and within that group, older adults, women, and Black patients are significantly less likely to receive a referral [9]. Women are 36 percent less likely to enroll in CR compared with men, with participation rates for non-Hispanic Black, Hispanic, and Asian women falling between 10 and 12 percent [42]. Among Medicare beneficiaries with HF, men showed significant increases in CR participation following the 2014 coverage expansion, rising from 2.8 to 6.4 percent, while women showed no significant improvement and maintained flat utilization around 2 to 4 percent [8]. Only 13.6 percent of non-Hispanic Black patients, 13.2 percent of Hispanic patients, and 6.9 percent of dual Medicare-Medicaid eligible patients participate in CR [5].

Women are more likely than men to report caregiving responsibilities as barriers to attendance [42]. Financial constraints also matter: lack of adequate insurance coverage, high copayments, and inability to take time off work prevent participation for many underinsured patients [13]. Inconvenient scheduling, exercise formats that feel unfamiliar or unappealing, lack of culturally sensitive programming, digital access limitations, and low health literacy compound these challenges [12,15,18].

### Practical Mitigations

Automatic or electronic referral systems combined with assisted enrollment and inpatient liaison programs are the most impactful upstream interventions. Automatic referral has shown up to a 10-fold increase in referral rates and, combined with case management, up to a 25-fold increase for women specifically [15,42]. Dedicated navigators or case managers address downstream barriers including transportation assistance, insurance authorization support, and coordination around competing responsibilities [12,13]. Hybrid and home-based CR models show promise for bridging access gaps; home-based initiation rates reach 43 percent compared with 13 percent for center-based programs in HF populations [22]. Equity metrics should be tracked as standard program indicators, with quality improvement cycles targeting disparities by race, sex, age, rurality, and socioeconomic status. The Million Hearts Cardiac Rehabilitation Collaborative has set an enrollment target of 70 percent for eligible patients, a benchmark that cannot be reached without explicitly equity-focused implementation [4].

## 12. Practical Synthesis: What a Modern HF-CR Program Should Look Like

A contemporary HF-CR program can no longer be built around a single, uniform protocol. The heterogeneity of HF demands a multidisciplinary, phenotype-informed approach that matches the intensity, modality, and clinical focus of rehabilitation to each patient’s specific profile.

For younger, clinically stable HFrEF patients, both HIIT and moderate continuous aerobic exercise produce meaningful gains in peak VO2 and LVEF, whether delivered in a center or at home, and rehabilitation works best when paired with rigorous GDMT optimization and systematic cardiovascular risk factor management [6,21]. For HFpEF with a metabolic or obese phenotype, combined aerobic and resistance training integrated with weight loss strategies and management of diabetes, hypertension, and obesity produces improvements in both exercise capacity and quality of life [11,30]. Older or frail HFpEF patients require lower-intensity, function-oriented programs centered on balance, gait training, strength, and comprehensive geriatric assessment, complemented by nutritional support and psychosocial care [35]. For post-hospitalization patients of any ejection fraction, early step-down CR initiated close to discharge and transitioned to outpatient or telerehabilitation provides the structure needed for rapid functional recovery and safe medication up-titration [37].

The REHAB-HF trial demonstrated a 1.5-point improvement in SPPB score, exceeding the minimal clinically important difference of 0.5 points threefold, alongside meaningful gains in six-minute walk distance (34 m), health status (7.1 KCCQ Overall Summary points), and depression scores [37]. All-cause rehospitalization at 6 months, a prespecified secondary outcome, did not differ significantly between groups (rate ratio 0.93; 95% CI 0.66 to 1.19) and there was no mortality benefit, so the trial should be interpreted as demonstrating benefit on physical function, health status, and depressive symptoms rather than as evidence for reduction in rehospitalization risk [37]. Frail patients showed 2.6-fold larger improvements in SPPB than prefrail patients, directly challenging the assumption that frailty limits rehabilitation responsiveness [35].

### A Minimal Core Outcome Set for Every HF-CR Program

Patient-reported health status is best captured using the KCCQ-12, assessed at baseline and at 3 and 6 months, with a clinically meaningful change defined as a 5-point improvement [2,24]. Functional performance should be measured through six-minute walk distance (minimum clinically important difference [MCID]: 30 m), the SPPB for older or frail patients (MCID: 0.5 points), and peak VO2 where CPET is available [20,35]. Program utilization should be tracked against the Million Hearts national participation goal of at least 70 percent of eligible patients attending one or more sessions and the AACVPR adherence measure of at least 12 completed sessions [43,44]. Programs should also monitor the higher pragmatic benchmarks proposed within the HF cardiac rehabilitation literature (session attendance of roughly two-thirds of prescribed visits and course completion above 80 percent), while recognizing that these thresholds are aspirational targets rather than validated performance measures [8,20]. Healthcare utilization (all-cause and HF-specific hospitalizations, emergency department visits, and mortality) rounds out the core set [45]. Supplementary measures including the PHQ-9 for depression screening, frailty assessment using the Fried criteria or Clinical Frailty Scale, and NYHA functional class add important clinical context for older and post-hospitalization populations.

Regardless of phenotype or clinical setting, every HF-CR program should incorporate a thorough baseline assessment from which an individualized, monitored exercise prescription is developed, nutritional counseling and medication review running in parallel, psychosocial assessment and intervention as standard components, continuous risk factor management, and structured patient education on self-care and symptom recognition, all underpinned by clear, ongoing communication with the referring provider and the broader HF care team.

## 13. Conclusions and Future Directions

CR is a multicomponent, guideline-endorsed intervention whose benefits in HFrEF are robust and consistent. Improvements in peak VO2, health-related quality of life, and hospitalization risk are reliably demonstrated in meta-analyses, individual participant data analyses, and large observational cohorts, and these benefits scale with the dose actually received [6,7,14,21].

The referral and enrollment pipeline in HFrEF is broken at multiple identifiable points, and the patients least likely to complete it carry the greatest burden of disease [8,9,12,18]. The tools to fix this already exist: automatic EHR-triggered referral, patient navigation, early appointment scheduling, and flexible delivery options. What has been lacking is systematic adoption.

HFpEF represents the most consequential coverage and evidence gap in contemporary CR. HFpEF and HFmrEF together account for the majority of HF cases and both remain outside Medicare coverage [10,11]. Available trial evidence consistently shows functional and quality-of-life benefits with supervised exercise, and the 2023 AHA/ACC scientific statement endorsed its clinical utility, but a large pragmatic trial aligned with coverage endpoints has not yet been conducted [19,30,31,32]. Conducting such a trial, with a pragmatic design that enrolls patients from community and safety-net settings, uses transparent dose reporting aligned with standard CR program structures, specifies KCCQ and functional capacity as co-primary endpoints, and includes hybrid and telehealth delivery arms, should be the single highest research priority in HF-related CR.

Delivery model diversification is both achievable and necessary. Telehealth and hybrid CR demonstrate safety, high adherence, and short-term functional and health-status outcomes comparable to center-based programs for appropriately selected, clinically stable HF patients, though evidence for hard clinical endpoints and long-term durability remains more limited and warrants continued study [22,27,43]. The 2026 CMS Physician Fee Schedule Final Rule provides permanent regulatory support for virtual direct supervision of CR, intensive CR, and pulmonary rehabilitation, and adds CR codes to the Medicare telehealth list for office-based programs [16]. However, the authority for HOPD-based programs to deliver virtual CR to a patient’s home remains temporary under the Consolidated Appropriations Act of 2026 and is set to expire on December 31, 2027 absent further congressional action [17]. Outside the United States, cardiac telerehabilitation has been integrated into national quality frameworks in Europe and adopted at scale in several single-payer systems, indicating that the operational and clinical case for virtual delivery is not U.S.-specific even as reimbursement architecture is [43]. Securing durable HOPD telehealth authority should be a near-term policy priority for U.S. cardiovascular professional societies.

For HFrEF, where efficacy is established, the critical gaps are in translation from evidence to practice. EHR-embedded clinical decision support tools evaluated through stepped-wedge cluster trials across diverse health systems, and patient navigation models employing community health workers or peer navigators tested with equity metrics as co-primary outcomes, are high-impact implementation priorities [8,9,12,42].

Equity cannot be treated as secondary. Disparities in CR access by sex, race, socioeconomic status, and geography are well documented, structurally embedded, and not closing on their own [12,13,42]. Coverage expansion, implementation investment, and delivery model innovation must each be designed explicitly to reduce these gaps. Research priorities include evaluation of copayment waiver programs and bundled payment models, concurrent evaluation of digital literacy training and technology provision as CR co-interventions, and federally supported demonstration projects to establish satellite or mobile CR capacity in rural and safety-net settings [13,22]. If this agenda is pursued with appropriate rigor, the next generation of HF and CR guidelines may describe CR as a routine, equitable component of HF care across the full ejection fraction spectrum.

**Call to action:** Realizing the full potential of CR in HF will require a coordinated multifaceted strategy encompassing four priorities; expansion of CMS coverage to stable HFpEF based on emerging trial evidence; widespread adoption of automated referral mechanisms and navigator-led enrollment for HFrEF; incorporation of hybrid, telehealth CR modalities alongside legislative efforts to make HOPD home-based delivery authority permanent before its scheduled 2027 sunset; and embedding equity-focused principles throughout the care continuum.

## Figures and Tables

**Table 1:** Cardiac Rehabilitation Delivery Models.

Model	Supervision	Key Features
Center-based	Direct, in-person	Reference standard; telemetry available; full multidisciplinary team present
Hybrid	Partial (in-person plus remote)	Flexible scheduling; maintains face-to-face contact; remote monitoring between sessions
Virtual/synchronous telehealth	Remote, real-time	Clinician-supervised via video; requires home technology access
Home-based/remote	Asynchronous or minimal	Maximum access; requires robust monitoring and escalation protocols

## Data Availability

Not applicable. No new data were created or analyzed in this study.

## Abbreviations

6MWT/6MWD, Six-Minute Walk Test/Distance; AHA, American Heart Association; ACC, American College of Cardiology; AACVPR, American Association of Cardiovascular and Pulmonary Rehabilitation; AF, Atrial Fibrillation; ARNI, Angiotensin Receptor-Neprilysin Inhibitor; CAA, Consolidated Appropriations Act; CI, Confidence Interval; CMS, Centers for Medicare and Medicaid Services; CPET, Cardiopulmonary Exercise Testing; CR, Cardiac Rehabilitation; CRT, Cardiac Resynchronization Therapy; EHR, Electronic Health Record; ESC, European Society of Cardiology; FITT-VP, Frequency, Intensity, Time, Type, Volume, Progression; GDMT, Guideline-Directed Medical Therapy; HF, Heart Failure; HFpEF, Heart Failure with Preserved Ejection Fraction; HFmrEF, Heart Failure with Mildly Reduced Ejection Fraction; HFrEF, Heart Failure with Reduced Ejection Fraction; HFSA, Heart Failure Society of America; HIIT, High-Intensity Interval Training; HOPD, Hospital Outpatient Department; HRQoL, Health-Related Quality of Life; ICD, Implantable Cardioverter-Defibrillator; KCCQ, Kansas City Cardiomyopathy Questionnaire; LVAD, Left Ventricular Assist Device; LVEF, Left Ventricular Ejection Fraction; MCID, Minimal Clinically Important Difference; MLHFQ, Minnesota Living with Heart Failure Questionnaire; MRA, Mineralocorticoid Receptor Antagonist; NCD, National Coverage Determination; NNT, Number Needed to Treat; NYHA, New York Heart Association; PFS, Physician Fee Schedule; PHQ-9, Patient Health Questionnaire-9; PRO, Patient-Reported Outcome; QI, Quality Improvement; RCT, Randomized Controlled Trial; RPE, Rating of Perceived Exertion; RR, Relative Risk; SGLT2, Sodium–Glucose Cotransporter-2; SMD, Standardized Mean Difference; SPPB, Short Physical Performance Battery; VE/VCO2, Ventilatory Efficiency Slope; VO2, Volume of Oxygen Consumption.
